# Bioleaching of Heavy Metals from Printed Circuit Boards with an Acidophilic Iron-Oxidizing Microbial Consortium in Stirred Tank Reactors

**DOI:** 10.3390/bioengineering9020079

**Published:** 2022-02-16

**Authors:** Juan Tapia, Alex Dueñas, Nick Cheje, Gonzalo Soclle, Nila Patiño, Wendy Ancalla, Sara Tenorio, Jorge Denos, Homar Taco, Weiwei Cao, Diogo A. M. Alexandrino, Zhongjun Jia, Vitor Vasconcelos, Maria de Fátima Carvalho, Antonio Lazarte

**Affiliations:** 1 Laboratorio de Biotecnología Ambiental, Biominería y Bioensayos Ecotoxicológicos (LAB-BIOTBEC), Escuela Profesional de Biología, Facultad de Ciencias Biológicas, Universidad Nacional de San Agustín, Arequipa 04000, Peru; jtapiacru@unsa.edu.pe (J.T.); aduenasg@unsa.edu.pe (A.D.); ncheje@unsa.edu.pe (N.C.); gsoclle@unsa.edu.pe (G.S.); npatinof@unsa.edu.pe (N.P.); wancalla@unsa.edu.pe (W.A.); stenorio@unsa.edu.pe (S.T.); denosadk@hotmail.com (J.D.); htaco@unsa.edu.pe (H.T.); alazarter@unsa.edu.pe (A.L.); 2 State Key Laboratory of Soil and Sustainable Agriculture, Institute of Soil Science, Chinese Academy of Sciences, Nanjing 210008, China; wwcao@issas.ac.cn (W.C.); jia@issas.ac.cn (Z.J.); 3 Interdisciplinary Centre of Marine and Environmental Research (CIIMAR), Terminal de Cruzeiros de Leixões, AV. General Norton de Matos, s/n, 4450-208 Matosinhos, Portugal; dalexandrino@ciimar.up.pt (D.A.M.A.); vmvascon@fc.up.pt (V.V.); 4 Faculty of Sciences, University of Porto, Rua do Campo Alegre, 4169-007 Porto, Portugal; 5 School of Medicine and Biomedical Sciences (ICBAS), University of Porto, Rua de Jorge Viterbo Ferreira 228, 4050-313 Porto, Portugal

**Keywords:** printed circuit boards, heavy metals, acidophilic iron-oxidizing bacterial consortium, metals tolerance, bioleaching

## Abstract

In this study, bioleaching was carried out for the recovery of metals (copper, zinc, tin, lead, gold and silver) from printed circuit boards residues (PCBs), one of the most important wastes from electrical and electronic equipment, using an acidophilic iron-oxidizing bacterial consortium enriched with minerals from a gold mine in the Arequipa region, Peru. High-throughput sequencing and analysis of the 16S rRNA biomarker revealed that this consortium was predominantly composed of *Tissierella, Acidiphilium* and *Leptospirillum* bacteria, from which the latter is known to grow by chemolithotrophy through iron oxidation. After the enrichment process, the acidophilic iron-oxidizing consortium was first tested for its tolerance to different PCBs concentrations, showing best growth up to 10 g/L of PCBs and a tolerance index of 0.383. Based on these results, the bioleaching efficiency of the consortium was investigated for 10 g/L of PCBs in stirred tank reactors coupled to an aeration system, for 18 days. High bioleaching efficiencies were achieved for copper and zinc (69% and 91%, respectively), indicating that these two metals can be easily extracted in this leaching system. Lower extraction efficiencies were achieved for tin (16%) and gold (28%), while for lead and silver only a residual recovery (<0.25%) was detected. These results indicate that the enriched bacterial consortium originating from the Arequipa region, Peru, has a high capacity to recover different metals of economic importance.

## 1. Introduction

The growing demand for electrical and electronic equipment (EEE) has been fueled by the prosperous growth of societies. This, coupled with the short lifespan of these devices has led to the generation of increasing levels of electrical and electronic equipment waste (WEEE). The lack of safety in their treatment and disposal, such as their incineration in the open air or introduction into landfills, poses significant risks to the environment and human health [1,2]. In 2016, Asia generated the highest volume of e-waste (18.2 Megaton, MT), followed by Europe (12.3 MT), America (11.3 MT), Africa (2.2 MT) and Oceania (0.7 MT) [2]. The improper management of EEEW is a global environmental problem due to the presence of a wide variety of toxic substances, which include polycyclic aromatic hydrocarbons and dioxins, embedded in the devices [3,4]. Printed circuit boards (PCBs) are used in almost all electronic equipment, from portable equipment (mobile phones, iPads, toys, etc.) to large-scale equipment (televisions, computers, vehicles, etc.) [5]. PCBs are a complex mixture composed of 30% plastic, 40% ferrous and non-ferrous metals, and 30% silicates and inert oxides [6,7], and their weight varies depending on the source: 2% of the total weight for large electronic devices, 11% for laptops and 22% for mobile phones [8]. Therefore, efficient recycling is necessary through innovative strategies, but this is still quite limited due to the heterogeneity and complexity of the materials present in PCBs [9].

The pulverization of PCBs is a prerequisite for the liberation of metallic and non-metallic constituents, however, this process can cause a loss of up to 40% of precious metals and the formation of dangerous fine metallic powders, which contain brominated flame retardants, phenols and dioxins [10,11]. After pulverization, the solids obtained from PCBs are treated by pyrometallurgical, hydrometallurgical and biohydrometallurgical methods. Pyrometallurgical processes are mainly based on smelting technologies that require temperatures between 300 and 900 °C [12], but they are costly, demand high energy consumption and release toxic fumes (e.g., dioxins) to the environment [13]. On the other hand, hydrometallurgical methods use various inorganic solutions for the dissolution of metals, mainly sulfuric acid, hydrochloric acid and nitric acid [13]. The use of these methods has increased due to lower emissions of toxic gases (dioxins and phenols), the generation of less powder, the requirement of less energy and a significant recovery of metals, however, they generate acid drains that are highly polluting to the environment [14,15]. In contrast, biohydrometallurgy has been gaining interest due to the fact that it is environmentally less polluting and also has the potential to reduce operating costs and energy demand [13]. During this process, different chemolithoautotrophic microorganisms are used in bioleaching applications due to their ability to facilitate metals dissolution through a series of bio-oxidation reactions [16,17,18]. In this case, iron has a central role as an electron carrier. The oxidized form of iron, ferric ion (Fe^3+^), generated from the microbial oxidation of ferrous iron (Fe^2+^) compounds, acts as an oxidizing agent that is able to oxidize metal sulfides, after which it is chemically reduced to ferrous ion [19].

In recent decades, biohydrometallurgical strategies have gained increasing prominence for the recovery of metals. Biological approaches allow solving main limitations in pyrometallurgical methods in terms of high-temperature demand [20], also avoiding the risks posed by different dangerous acids used in hydrometallurgical methods. Many research works can be found evaluating the capacity of acidophilic bacteria for the extraction of metals from PCBs, mainly using *Acidithiobacillus thiooxidans*, *Acidithiobacillus ferrooxidans* and *Leptospirillum ferrooxidans*, constituting a sustainable alternative for the recovery of metals. High recovery efficiencies, more than 94% for copper (Cu) and 90% for zinc (Zn) were achieved with these microorganisms for PCB concentrations of 10 to 50 g/L and a leaching time of 4 to 10 days [5,20,21,22,23] (. On the other hand, cyanogenic bacteria (*Chromobacterium violaceum*, *Pseudomonas fluorescens* and *Pseudomonas aeruginosa*) were shown to be involved in gold and silver leaching processes, with a recovery efficiency of 65% and 30%, for solids concentration of 5 to 15 g/L [24,25,26,27]. The use of a bacterial consortium instead of single microorganisms can be more advantageous for metals bioleaching as different microorganisms can have distinct metals tolerance, which can result in a greater bioleaching capacity [28]. Overall, biological technologies have been attracting more interest due to the fact that they have the potential to cause a lower environmental impact, are easier to operate and have better cost-effectiveness than non-biological leaching approaches. Thus, the use of microorganisms in metal recycling is rapidly emerging as a greener technology, compared to smelting or chemical processing, while also proving useful for resource recovery and pollution mitigation [29]. Furthermore, bioleaching processes can ensure greater selectivity in the extraction of metals, a characteristic that is not easily achieved with chemical-based leaching approaches (e.g., hydrometallurgical methods), while also avoiding the use of acidic leaching solutions with harmful impacts to the environment [1]. The kinetics of these processes pose as their main disadvantage, though they can be substantially improved if bioleaching is carried out by microorganisms during their exponential growth phase and if they are maintained in optimal conditions of pH, temperature and aeration.

To achieve high bioleaching efficiency in e-waste metal extraction processes, one needs to control various physicochemical, microbiological and mineralogical factors. Stirred tank reactors allow good control of a wide range of parameters, such as temperature, pH, aeration, agitation among others, while at the same time providing high mass transfer, high mixing and a continuous bubble column, which renders the process of metal recovery much faster and more efficient [19,30,31].

In this work, we studied metal recovery from PCBs waste with an acidophilic iron-oxidizing bacterial community native to the Arequipa region, Peru, in stirred tank reactors coupled to an aeration system. Initially, tolerance of the acidophilic iron-oxidizing bacterial community to the heavy metals Cu and Zn were determined. After this, adaptation and tolerance tests were carried out with PCBs residues to determine the optimum growth concentration and obtain the greater efficiency of metal recovery in the reactors. This work investigated for the first time the capacity of an acidophilic iron-oxidizing bacterial consortium native to the Arequipa region, in Peru, to bioleach PCBs residues.

## 2. Materials and Methods

### 2.1. Preparation and Characterization of Printed Circuit Boards (PCBs)

The waste PCBs used in this study were obtained from Comimtel Recycling, Peru. The preparation of waste PCBs was carried out as follows: (i) manually separated electronic components (for example, capacitors, cards, batteries, resistors, among others) were size reduced by using metal cutting scissors and a portable powder crusher (Keene Engineering, Chatsworth, CA, USA), until obtaining a fine powder with a particle size ≤ 300 µm [32,33]; (ii) powder samples were then washed with a saturated solution of NaCl 35% (*w*/*v*), at a ratio of 10 g/100 mL, and were dried in an oven at 60 °C for 24 h (for the elimination of plastic particles potentially toxic to bacterial metabolism) [5,34].

For the characterization of waste PCBs metal content, the powder obtained was digested with an acid mixture of HNO_3_, HCl, HF and HClO_4_ in a ratio of 5:1:2:2, respectively [35]. The aforementioned digested solution was analyzed by Atomic Absorption Spectrophotometry (AAS) (AAnalyst100-Perkinelmer, Waltham, MA, USA), while the bioleaching solutions were analyzed by Inductively Coupled Plasma Optical Emission Spectrophotometry (ICP-OES) (Nexion350D-Perkinelmer, Waltham, MA, USA).

### 2.2. Composition of Culture Media

The culture media used in this study were 9 K medium and 4.5 K medium. The 9 K medium was composed of solutions A and B. Solution A (700 mL) had the following composition: 3 g (NH_4_)_2_SO_4_, 0.5 g K_2_HPO_4_, 0.5 g MgSO_4_·7H_2_O, 0.1 g KCl and 0.01 g Ca(NO_3_)_2_. This solution was sterilized by autoclaving at 121 °C for 20 min. Solution B (300 mL) consisted of 44.22 g FeSO_4_·7H_2_O and was sterilized with a 0.22 µm PTFE filter. Both solutions were combined to obtain 1 L of 9 K medium and pH was adjusted to 1.8–2.0 with 98% H_2_SO_4_ [36]. The 4.5 K medium had the same composition as the 9 K medium but with half the concentration in all salts.

### 2.3. Enrichment of Acidophilic Iron-Oxidizing Microbial Consortium

Seven solid samples along with tailing were collected from the Century Mining gold mine in the Arequipa region, Peru (15°54.931′ S; 73°2.845′ W). The solid samples were ground and pulverized to release the bacteria present, then 20 g of the resulting samples were added to a 250 mL capacity flask containing 150 mL of 9 K culture medium at an initial pH of 2. Cultures were set up in duplicate, incubated in an orbital shaker at 150 rpm, at a temperature of 28 °C, for 46 days. During the incubation period, the color of one of the cultures gradually changed from a pale green to a deep red, showing the existence of acidophilic iron-oxidizing bacteria in the culture. This bacterial consortium was used to reseed (10% inoculum) 100 mL flasks containing 40 mL of 9 K medium that were incubated at 30 °C in an orbital shaker at 150 rpm, until the culture turned reddish due to the oxidation of the ferrous ion (Fe^2+^) to ferric ion (Fe^3+^). This reseeding procedure was repeated for 9 weeks until organisms completely adapted to the 9 K medium were obtained.

### 2.4. Bacterial Profiling of the Enriched Acidophilic Iron-Oxidizing Consortium by High-Throughput Sequencing of the 16S rRNA Amplicon

Aliquots of the acidophilic iron-oxidizing consortium were centrifuged at 12,000 rpm for 15 min and the obtained pellets were resuspended in ethanol 70% (*v*/*v*). Bacterial DNA was isolated using E.Z.N.A.^®^ Bacterial DNA Kit (Omega Bio-Tek, Inc., Norcross, GA, USA) according to the manufacturer’s recommendations. 

Illumina Miseq platform (Illumina Inc., San Diego, CA, USA) was employed for the high-throughput sequencing of the 16S rRNA gene. Amplification by PCR was carried out using the universal bacterial primers pair 515F/907R, which targets the V4-V5 hypervariable region of the bacterial 16S rRNA gene. The primers pair has a 12 bp barcode at the forward primer to identify different samples. The reaction (total volume, 50 µL) consisted of 2 μL of DNA template (DNA concentration: 2–8 ng μL^−1^), 1 μL of each forward/reverse primer (each 10 μM), 25 μL of SYBR Premix Ex TaqTM (Tli RNaseH Plus, TaKaRa, Kusatsu, Japan), and 21 μL of sterilized distilled water. The PCR thermal profile for amplifying the 16S rRNA gene consisted of an initial denaturation step at 94 °C for 2 min, followed by 30 cycles of 94 °C for 30 s, 60 °C for 30 s, and 72 °C for 45 s [37]. PCR products were purified after 1.2% agarose gel electrophoresis to confirm amplicon size and specificity (single band) and subsequently mixed at equimolar concentrations for Illumina MiSeq sequencing. A sequencing library was constructed for each gene using the TruSeq Nano DNA LT Sample Prep Kit Set A (Illumina Inc., San Diego, CA, USA), and the sequencing was performed with the MiSeq Reagent Kit v3 (Illumina Inc., San Diego, CA, USA) (600 cycles).

The 16S rRNA dataset was analyzed using the QIIME (Quantitative Insights Into Microbial Ecology) pipeline [38]. The scripts and instructions of the QIIME pipeline are publicly available at http://qiime.org, accessed on 12 December 2021. Briefly, sequences were first joined by the command “join_paired_ends.py”, then barcodes were extracted by “extract_barcodes.py”, then trimmed and demultiplexed by “split_libraries_fastq.py” at Phred quality score of 25. Chimeras were identified and removed using the command “identify_chimeric_seqs.py” in usearch61 [39]. The 16S rRNA gene sequences were classified and clustered into Operational Taxonomic Units (OTUs) at the 97% identity threshold, by using “pick_open_reference_otus.py”. Non-bacterial OTUs were removed from the dataset and a limit relative abundance threshold of 1% was applied for all taxonomic ranks, using the phyloseq package in R environment (version 3.6.1) [40]. 

### 2.5. Tolerance of the Enriched Acidophilic Iron-Oxidizing Consortium to Metals and PCBs Waste

Tolerance experiments were carried out in 100 mL flasks with 40 mL of 9 K culture medium adjusted to pH 1.8 and supplemented with metals (Cu or Zn) or waste PCBs. The flasks were inoculated with 10% (*v*/*v*) of the acidophilic iron-oxidizing consortium previously enriched. Cultures were incubated with shaking at 150 rpm in an orbital shaker and at a temperature of 30 °C. Control experiments were also carried out under the same conditions but without the addition of metals or PCBs waste. All experiments were carried out in triplicate.

#### 2.5.1. Tolerance to Cu and Zn

Each culture was supplemented with the concentrations of 10.5, 13, 18, 23, 28, 33 and 38 g/L of Cu (Cu_2_SO_4_) and Zn (ZnSO_4_). Metals were added to the cultures at the beginning of the experiments. Cultures were monitored for 7 days through the analysis of bacterial growth by counting bacterial cells in a Neubauer chamber with a phase-contrast microscope (Carl Zeiss Microscopy GmbH, Munich, Germany). Based on these results, inhibitory concentration (IC) and tolerance index (TI) were determined. IC is defined as the concentration in which no bacterial growth in the presence of the tested metals is obtained [41]. TI was calculated through the analysis of the bacterial growth rate in the presence of metals and in the control not containing metals, according to Equation (1) [42].

#### 2.5.2. Tolerance to PCBs Waste

To investigate the tolerance to PCBs waste, the acidophilic iron-oxidizing consortium previously enriched was initially adapted to PCBs waste through the serial acclimation method. After 2 days of growth, each culture developed a reddish coloration due to the oxidation of iron by the acidophilic iron-oxidizing consortium. At this stage, PCBs waste was introduced at a concentration of 2.5 g/L initiating the adaptation for 4 days, then the same procedure was carried out but with a concentration of 5 g/L. After the acclimation period with PCBs waste, tolerance experiments were carried out with concentrations of 5, 10, 15 and 20 g/L of PCBs that were added at the beginning of the experiment. Bacterial growth was monitored for 6 days through the counting of bacterial cells in a Neubauer chamber with a phase-contrast microscope (Carl Zeiss Microscopy GmbH, Munich, Germany). At the end of the experiment, TI was determined according to Equation (1).

(1) $$\mathrm{TI}=\frac{\mathrm{Cell}\;\mathrm{density}\;\left(\frac{\mathrm{bacteria}}{\mathrm{mL}}\right)\mathrm{with}\;\mathrm{Metals}\;\mathrm{or}\;\mathrm{PCBs}}{\mathrm{Cell}\;\mathrm{density}\;\left(\frac{\mathrm{bacteria}}{\mathrm{mL}}\right)\mathrm{without}\;\mathrm{Metals}\;\mathrm{or}\;\mathrm{PCBs}}$$

### 2.6. Bioleaching Experiments of PCBs

Bioleaching experiments were carried out in stirred tank reactors, consisting in cylindrical transparent glass tanks of 5 L capacity with 3 L of working volume, which contained a central axis with 2 Rushton impellers made of stainless steel 316. The reactors were placed on a cooking resistance attached to a digital pyrometer to maintain a constant operating temperature and were aerated by diffusing air through a ring-type bubble diffuser after passing it through a PTFE microfilter (0.22 μm) to eliminate bacteria and powder particles.

**Figure 1:**
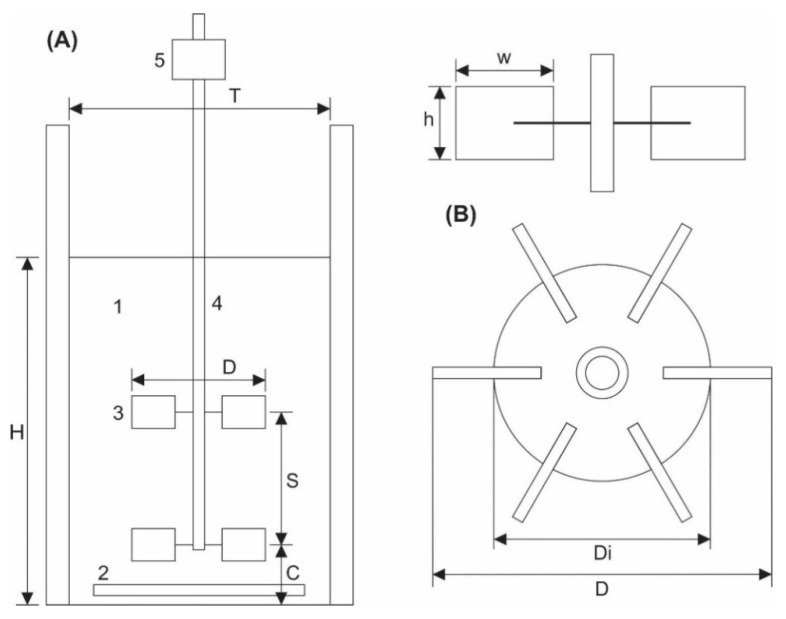
(**A**) Schematic representation of the stirred tank reactor (1. stirred tank, 2. ring-type bubble diffuser, 3. Rushton impeller, 4. Axis and 5. Rotation sensor), with the geometric proportions (H/T = 1.35, D = T/2, C = T/4, S = T/2). (**B**) Rushton impeller sketch with six blades to shaft (w/D = 1/3, h/D = 1/4, Di/D = 2/3). Schematics were adapted from Luan et al. [45].

Figure 1 shows the stirred tank reactor with height (H)/diameter (T) of 1.35. Rushton impellers with diameter (D) of T/2, were formed by 6 blades parallel to the axis of agitation with a length (w) of D/3 and a width (h) of D/4. The first impeller was located on a bottom free space (C) of T/4 and the distance between impellers (S) was T/2.

Each reactor contained 3 L of 4.5 K culture medium adjusted to pH 2 with H_2_SO_4_ (98%) to facilitate the availability of Fe^2+^ in the solution [43,44], and was inoculated with 10% (*v*/*v*) of the acidophilic iron-oxidizing consortium previously enriched. After 5 days of growth, iron oxidation was observed in each reactor and bacterial density achieved a value of 1.1 × 10^7^ bacterial cells/mL. At this point, 10 g/L of sterile powder of PCBs waste were added to each reactor to start the bioleaching experiments. The reactors were monitored for 18 days with a constant temperature of 30 ± 1 °C, a stirring of 150 rpm and an air flow of 500 mL/min. Microbial growth was evaluated through the analysis of the number of viable bacteria. pH and oxidation-reduction potential (ORP) were analyzed to evaluate microbial activity related with the bioleaching of metals present in WEEE and metal extraction was evaluated in the leaching solution to determine bioleaching efficiency (BE). This latter parameter was calculated, in percentage, taking into account the relationship between the concentration of metals recovered in the bioleaching solution and the total concentration of metals present in the waste PCBs (Equation (2)).

(2) $$\mathrm{BE}=\frac{\mathrm{Concentration}\;\mathrm{of}\;\mathrm{metals}\left(\frac{\mathrm{mg}}{L}\right)\mathrm{bioleaching}\;}{\mathrm{Concentration}\;\mathrm{of}\;\mathrm{metales}\;\left(\frac{\mathrm{mg}}{L}\right)\mathrm{in}\;\mathrm{PCBs}}\;\times \;100\%$$

### 2.7. Analytical Methods

Oxidation-reduction potential (ORP) and pH were determined with a Multiparameter probe (Aquaread; Broadstairs, UK). The number of cells in the liquid phase was calculated by direct counting in a Neubauer counting chamber under a phase-contrast microscope (model Primostar, Carl Zeiss; Jena, Germany). The leaching solutions were periodically analyzed to determine the concentration of Cu, Zn, tin, lead, gold and silver, using AAS and ICP-OES [35]. Statistical analysis was performed with the IBM SPSS software (v.25, Armonk, NY, USA).

## 3. Results and Discussion

### 3.1. Composition of the PCBs Waste

Using chemical methods with acid digestion, 18 metals were identified in the analyzed PCBs waste (Table 1). The results show that Cu is the most abundant metal with 10.63%, followed by aluminum (Al) (>1%), barium (Ba) (>1%) and calcium (Ca) (>1%). Considerable amounts of iron (Fe) (0.93%) and tin (Sn) (0.9%) were also found, together with other precious metals that were present in small quantities such as silver (Ag) (0.07%) and gold (Au) (0.01%). These results are very similar to those obtained by Xia et al. [46]. Sulfur and rare earth elements were not detectable by the methods used and/or were present in trace concentrations, the reason why they were not included in Table 1. 

### 3.2. Enrichment of an Acidophilic Iron-Oxidizing Consortium

**Figure 2:**
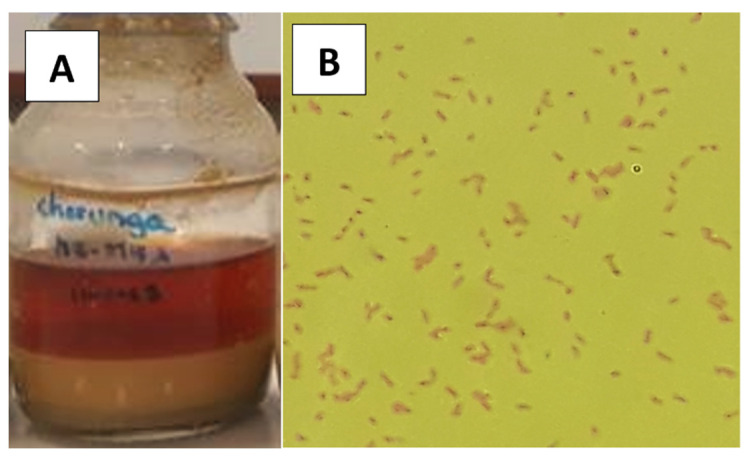
The acidophilic iron-oxidizing bacterial consortium enriched from a porous chalcopyrite mineral collected at Century Mining gold mine in the Arequipa region, Peru. (**A**) Bacterial culture. (**B**) Gram-negative bacteria observed under light microscopy.

From the seven cultures containing different minerals used for enriching acidophilic iron-oxidizing bacteria, only the one that contained a solid sample of a porous chalcopyrite mineral presented a reddish coloration, indicating the oxidation of ferrous ion (Fe^2+^) to ferric ion (Fe^3+^) in the 9 K medium (Figure 2). Phase-contrast microscopy (Carl Zeiss, Germany) of an aliquot of this culture revealed motile bacteria due to the presence of flagella, which were further identified as Gram-negative bacilli (determined by Gram staining). Subsequently, this consortium was enriched in a 9 K medium without the presence of mineral, achieving a turning time to the reddish color of 48 h.

### 3.3. Profiling of the Enriched Acidophilic Iron-Oxidizing Consortium by High-Throughput Sequencing of the 16S rRNA Amplicon

**Figure 3:**
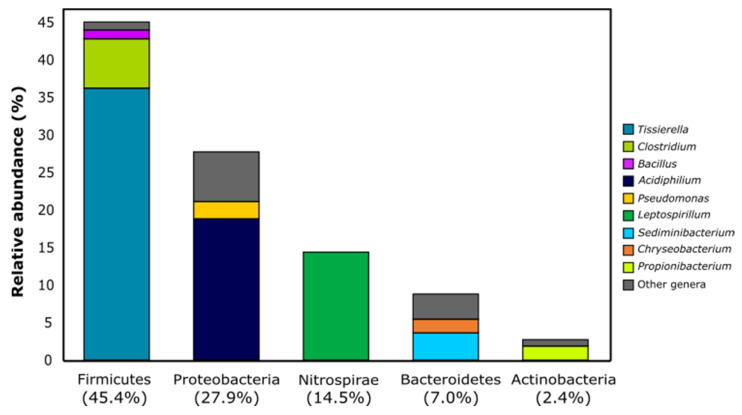
Bacterial composition of the enriched consortium based on the most dominant phyla and the main bacterial genera accommodated therein (>1% of relative abundance).

Bacterial profiling of the enriched acidophilic iron-oxidizing consortium revealed that approximately 90% of the community is dominated by the bacterial phyla Firmicutes (45.4%), Proteobacteria (27.9%) and Nitrospirae (14.5%) (Figure 3). The dominance of these taxonomical groups is mostly due to the abundance of one or two bacterial phylotypes belonging to each phylum. For Firmicutes, the genera *Tissierella* (36.6%) and *Clostridium* (6.6%) contribute almost entirely to the overall abundance of this phylum (Figure 3), while for Proteobacteria the same is observed due to the representation of *Acidiphillium* (19.0%) bacteria in the enriched consortium (Figure 3). For the Nitrospirae phylum, this contribution is even more evident, as its entire representation in the consortium is the result of the occurrence of *Leptospirillum* taxa (Figure 3). Although less represented, other bacterial genera were also detected in the consortium, including *Sediminibacterium* (3.6%), *Pseudomonas* (2.3%), *Propionibacterium* (1.9%), *Chryseobacterium* (1.9%) and *Bacillus* (1.2%). 

The enrichment process favorably selected iron-utilizing bacterial groups capable of thriving in acidophilic conditions, among which *Leptospirillum* and *Acidiphilium* stand out due to their high representation in the consortium. Their iron utilization pathways differ though, as *Leptospirillum* bacteria oxidize iron for chemolithotrophic growth [47], while *Acidiphilium* reduces this metal to stimulate heterotrophic growth [48,49]. Given the lack of heterotrophic substrates supplemented to the enrichment medium, the increased abundance of *Acidiphilium* may be partially explained by the favorable acidic conditions that could have provided them a competitive advantage against other bacterial taxa, while also being capable of oxidizing organic acids and cellular debris originating from vicinal microorganisms. The enriched consortium was also dominated by several Firmicutes, mostly the anaerobes *Tissierella* and *Clostridium,* which are not expected to participate in the bioleaching process. Instead, their occurrence may be linked to the high oxidation rates achieved by other members of the consortium, allowing the establishment of anoxic microenvironments. 

### 3.4. Tolerance of the Enriched Acidophilic Iron-Oxidizing Consortium to Cu and Zn

**Figure 4:**
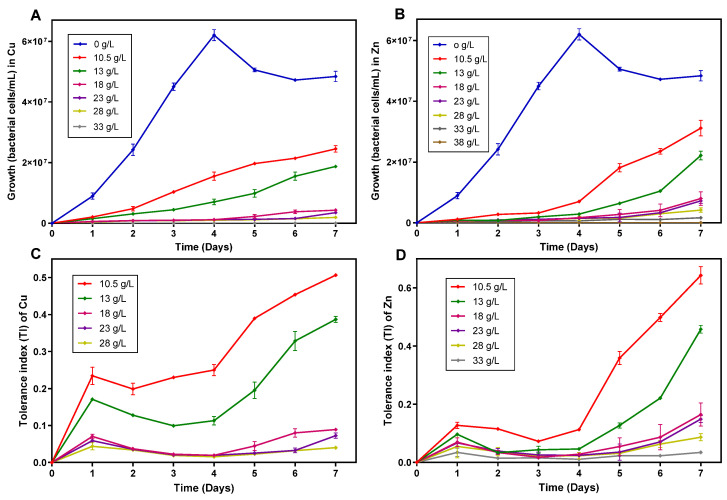
Growth and tolerance indices of the enriched acidophilic iron-oxidizing consortium in the presence of Cu (**A**,**C**) and Zn (**B**,**D**).

The tolerance of the iron-oxidizing acidophilic bacterial consortium to Cu and Zn salts was investigated since these metals are usually recovered more efficiently from PCBs waste [5,20,46]. The growth of the enriched acidophilic iron-oxidizing consortium at different concentrations of Cu and Zn (0–38 g/L) along an experimental period of 7 days, is shown in Figure 4. The growth of the bacterial consortium decreased with the increase in Cu and Zn concentration. The acidophilic iron-oxidizing culture was able to oxidize all the ferrous iron available in the 9 K medium in approximately 48 h when Cu and Zn were not added to the culture medium, however, in the presence of high concentrations of Cu (28 g/L) and Zn (33 g/L), cultures oxidized less than 10% of the available iron. Similar results were reported by other authors [50,51,52,53]. Nonetheless, it was also shown that microorganisms can overcome growth inhibition caused by Cu and Zn, and completely oxidize the ferrous iron available in the 9 K culture medium, with a prolonged incubation period of up to 300 h [52,54], due to the expression of several genes resistant to high concentrations of Cu and other heavy metals [55].

In the presence of Cu, the bacterial consortium achieved the highest growth at a concentration of 10.5 g/L, attaining a cell density of 2.5 × 10^7^ bacterial cells/mL after a period of 7 days. At Cu concentrations of 18 and 28 g/L, the bacterial consortium showed a long lag phase (6 days), only presenting a slight growth at the end of the experimental period of 7 days (Figure 4A), reaching bacterial densities of 4.3 × 10^6^ and 1.9 × 10^6^ bacterial cells/mL, respectively. In the presence of Zn, the bacterial consortium exhibited behavior similar to that observed with Cu (Figure 4B). The highest growth was obtained for a Zn concentration of 10.5 g/L, in which cell density achieved a value of 3.1 × 10^7^ bacterial cells/mL. For Zn concentrations ≥18 g/L, the bacterial consortium presented a long lag phase (5 days), reaching at the end of the 7-day experimental period bacterial densities between 8.0 × 10^6^ and 1.7 × 10^6^ bacteria/mL for 18–33 g/L of Zn.

The inhibitory concentration of Cu and Zn to the enriched bacterial consortium was 33 and 38 g/L, respectively. These inhibitory values are within the range of other values reported in the literature. Mangold et al. [56] reported Zn inhibitory concentrations of 52.3 and 49.1 g/L for *Acidimicrobium ferrooxidans* DSM10331 and *Ferroplasma acidarmanus* Fer1, respectively. Cabrera et al. [57] revealed that *Acidithiobacillus ferrooxidans* was able to tolerate up to 10 g/L of Cu and 50 g/L of Zn and Novo et al. [52] described an inhibitory concentration of 39.2 g/L of Zn for this microorganism, though it could tolerate concentrations greater than 40 g/L of Cu. Nevertheless, prolonged contact between heavy metals and microorganisms can cause microbial acclimatization; therefore, acclimatized microorganisms can eventually grow in concentrations of heavy metals significantly higher, compared to the same non-acclimatized microbial strains [50,51,55,57].

Figure 4C,D show the tolerance index (TI) of the enriched microbial consortium for Cu and Zn concentrations between 0 and 33 g/L. TI decreased with the increase in Cu and Zn concentrations. When these metals were added to the culture medium, the highest TI values of 0.507 and 0.643 were obtained at the concentration of 10.5 g/L of Cu and Zn, respectively. Two stages are shown in the TI curves at concentrations ≥13 g/L; (a) the lag phase that occurs during 4 days at the beginning of the process, with TI = 0.113 (Cu) and TI = 0.046 (Zn); (b) continuous rapid growth of the curves until day 7 with TI = 0.387 (Cu) and TI = 0.458 (Zn). 

### 3.5. Tolerance of the Enriched Acidophilic Iron-Oxidising Consortium to PCBs Waste

**Figure 5:**
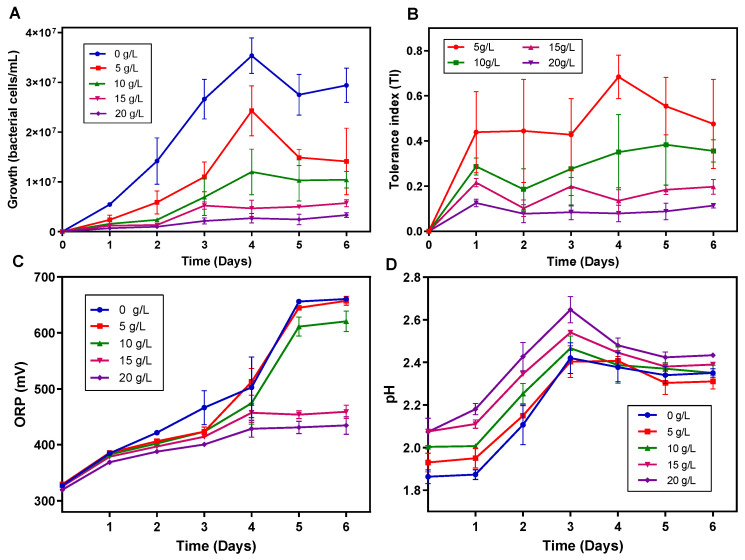
Growth (**A**), tolerance index (**B**), oxidation-reduction potential (ORP) (**C**) and pH (**D**) registered in the enriched acidophilic iron-oxidizing consortium cultures in the presence of PCBs waste.

Before testing the tolerance to PCBs waste, the enriched acidophilic iron-oxidizing consortium was acclimatized first with 2.5 g/L and after with 5 g/L of PCBs waste. After this step, cultures were supplemented with a range of concentrations of PCBs waste that varied between 5–20 g/L. Figure 5A shows that growth of the iron-oxidizing bacterial consortium in the presence of PCBs waste decreased with the increase in the concentration of PCBs waste, being also observed an increase in the respective growth lag phases. The highest growth was observed on day 4 for the concentrations of PCBs waste of 5 and 10 g/L, with a bacterial density of 2.4 × 10^7^ and 1.2 × 10^7^ bacteria/mL being respectively achieved. On the other hand, very slight growth was observed for the concentrations of 15 and 20 g/L, being obtained a bacterial density of 5.7 × 10^6^ and 3.3 × 10^6^ bacteria/mL, respectively. This was probably due to the inhibitory effect and toxicity of dangerous compounds such as heavy metals, plastic, phenols and dioxins present in PCBs dust [33]. 

Figure 5B shows the TI of the microbial consortium against PCBs waste with respect to the control culture. TI values decreased with the increase in PCBs concentration from 5 to 20 g/L. The highest TI values obtained were 0.684 (after 4 days) and 0.383 (after 5 days) for 5 and 10 g/L of PCBs, respectively, while at 15 and 20 g/L TI was lower than 0.20. Therefore, in the light of these results, we found it adequate to carry out bioleaching experiments using PCB concentrations up to 10 g/L. Similarly, Işıldar et al. [6] reported optimal growth of a consortium composed of *Acidithiobacillus ferrooxidans* DSM17398 and *Acidithiobacillus thiooxidans* DSM9463 at 10 g/L of PCBs, in experiments carried out with 5, 10, 25 and 50 g/L of waste PCBs. On the other hand, Yang et al. [58] adapted *Acidithiobacillus ferrooxidans* SW-02 up to 0.25 g/L of PCBs, after which tested the growth of this microorganism with PCBs concentrations of 15, 25 and 35 g/L, and found that the optimal concentration for leaching was 15 g/L. 

The variation of pH and ORP along the experiment is shown in Figure 5C,D. The redox potential is known to be influenced by metal reactivity, microbial activity and leaching conditions [46]. In our study, we anticipate that the aeration conditions, combined with the pH levels and incubation conditions of the enrichment cultures, were key drivers of the ORP fluctuations observed. Indeed, all PCBs concentrations showed an increase in the redox potential (Figure 5C), with the highest peaks being observed at day 6, possibly due to the oxidation of the metals in PCBs by Fe^3+^, concomitantly generated from the iron available as Fe^2+^ in abundance in the culture medium. Since the redox potential was positive, oxidation processes should have occurred in the system. Pourhossein and Mousavi [42] reported similar ORP values in an experiment of bioleaching of Light Emitting Diode (LED) with *Acidithiobacillus ferrooxidans* at 5, 10, 15, 20 and 25 g/L. In addition, the ORP values observed in the present study were also comparable to those obtained by Wu et al. [23] when bioleaching PCBs waste (0, 5, 10, 15 g/L) with a culture supernatant derived from a bacterial consortium predominantly constituted by *Leptospirillum ferriphilum* and *Sulfobacillus thermosulfdooxidans*. The pH showed a similar trend in all PCBs concentrations, increasing progressively until day 3, with maximum values of 2.65 and 2.54 for 20 and 15 g/L, respectively, followed by 2.47, 2.42 and 2.40 for 10, 0 and 5 g/L, respectively. Then, after day 6 the pH decreased slightly. In general, the results obtained indicate that when the consortium was supplemented with lower PCBs concentrations, lower pH values were also registered in the cultures. These pH oscillations observed in the cultures could have affected bacterial growth as pH values were not within the optimum range of 1.8–2.5 [43].

### 3.6. PCBs Bioleaching in Stirred Tank Bioreactors

**Figure 6:**
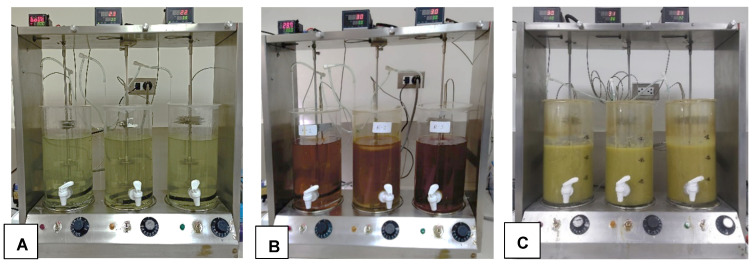
Bioleaching in stirred tank bioreactors coupled to an aeration system and operated at 30 ± 1 °C: (**A**) reactors with 4.5 K culture medium and inoculated with 10% (*v*/*v*) of the enriched acidophilic iron-oxidizing bacterial consortium; (**B**) bio-oxidation of the 4.5 K culture medium; (**C**) Bio-leaching solution.

Bioleaching was carried out in stirred tank reactors coupled to an aeration system, with a height/diameter of 1.35, very similar to the designs and methods of Luan et al. [45] and Srirugsa et al. [59]. Each tank contained 3 L of 4.5 K culture medium adjusted to an initial pH of 2, inoculated with 10% (*v*/*v*) of the enriched acidophilic iron-oxidizing bacterial consortium (Figure 6A). The reactors were operated at a temperature of 30 ± 1 °C, a stirring of 150 rpm and an air flow of 500 mL/min. After 5 days of bacterial growth, a reddish coloration of the culture medium was observed in each bioreactor due to the oxidation of Fe^2+^ to Fe^3+^, having achieved a density of 1.1 × 10^7^ bacteria/mL (Figure 6B). Subsequently, 10 g/L of waste PCBs with a particle size ≤ 300 µm were added to each bioreactor, and bioleaching was evaluated for 15 days for the metals Cu, Zn, tin, lead, gold and silver (Figure 6C).

The bioleaching efficiency of Cu, Zn, gold and tin from the PCBs waste by the bacterial consortium is shown in Table 2. The results showed that at a concentration of 10 g/L of powder of PCBs waste with a constant temperature of 30 ± 1 °C, a stirring of 150 rpm and an air flow of 500 mL/min, the adapted bacterial consortium achieved a sharp increase in the extraction of Zn and Cu at day 3, with an efficiency of 58.88% and 65.04%, respectively, and attained then the maximum extraction value of 91.36% and 68.55% for Zn (at day 15) and Cu (at day 11), respectively. These results indicate that Zn can be easily recovered using this leaching system, and are similar to those reported by Gu et al. [5], who obtained 90.78% recovery of this metal by *Acidithiobacillus ferrooxidans* from PCBs waste. However, our results are considerably better than that reported by Becci et al. [20], who was able to recover only 70% of Zn from PCBs waste by a consortium formed by *Acidithiobacillus ferrooxidans* and *Leptospirillum ferrooxidans*. In contrast, Cu presented a lower extraction efficiency, which was very close to the recovery of 74% with *Acidithiobacillus ferrooxidans*, reported by Gu et al. [32], but other studies indicate a higher bioleaching efficiency for this metal that ranges from 94% to 100% from PCBs waste [5,20,21,22]. Additionally, Ilyas et al. [60] performed an acid pretreatment to evaluate the microbial activity of *Sulfobacillus thermosulfidooxidans* under intense aeration, obtaining a recovery of 92% of Cu and 93% of Zn. The maximum extraction efficiency of gold achieved in this study was 27.9% on day 11, while tin extraction achieved a maximum value of only 15.6% on day 3. Işıldar et al. [6] reported recovery of 44% of gold from PCBs waste under alkaline conditions using cyanide-producing heterotrophic bacteria, indicating that bioleaching of this metal may be more efficient under alkaline conditions than under acidic conditions, like those used in the present study. On the other hand, tin bioleaching was reported to achieve 90.2% using a mixed culture of *Acidithiobacillus ferrooxidans* and *Acidithiobacillus thiooxidans* [61], in spite of others suggesting that tin was not bioleached but rather precipitated as SnO [18,62], therefore on day 7 a slight decrease in tin is observed. For silver and lead, a recovery efficiency below 0.25% was achieved in the present study. This low percentage of lead recovery may be a consequence of PbSO_4_ precipitation that results in a decrease in leaching [5,62,63]. In addition, gold and silver are mobilized more efficiently under alkaline conditions (pH 9) than at acidic conditions [24]. 

The enriched acidophilic iron-oxidizing bacterial consortium extracted the heavy metals composing the PCBs waste through the following steps: first, ferrous ions (Fe^2+^) are oxidized to ferric ions (Fe^3+^) for the consortium to derive energy; then, the produced ferric ions (Fe^3+^) oxidize the metals of the PCBs, as shown in Equations (3)–(6) at a working temperature of 30 ± 1 °C. These equations are general expressions. Specifically for Equation (5), gold dissolution can be efficiently carried out either with ferric chloride at 25–95 °C [64] or with ferric sulfate and acid thiourea at 25 °C [65]. The reaction described in this Equation, while not dominant at ambient temperature, has shown to be promoted with adequate cultivation conditions, namely with increasing temperatures [66]. Thus, the ferric ions produced by iron-oxidizing microorganisms play an important role in the recovery of metals [20,58,61]. Therefore, leaching was started in reactors containing the bacterial culture with a high concentration of Fe^3+^ to improve the extraction efficiency of metals present in PCBs (Figure 6B).

2Fe^3+^ + Cu^0^ → 2Fe^2+^ + Cu^2+^ (3)

2Fe^3+^ + Zn^0^ → 2Fe^2+^ + Zn^2+^ (4)

3Fe^3+^ + Au^0^ → 3Fe^2+^ + Au^3+^ (5)

4Fe^3+^ + Sn^0^ → 4Fe^2+^ + Sn^4+^ (6)

It is very important that the process for the regeneration of Fe^2+^ in the bioleaching reactions is cyclical (Equation (7)) to be able to maintain the extraction of heavy metals. It is also reasonable to assume that part of the zero valence metals may have been directly chemically or biologically leached, according to Equations (8) and (9), with the resulting hydroxide ions being responsible for the increase in pH during the leaching process [42,43].

Fe_2_(SO_4_)_3_ + M^0^ → M^2+^ + SO_4_^2−^ + 2FeSO_4_ (7)

4M^0^ + 12H^+^ + 3O_2_ → 4M^3+^ + 6H_2_O (8)

2M^0^ + 2H_2_O + O_2_ → 2M^2+^ + 4OH^−^ (9)

**Figure 7:**
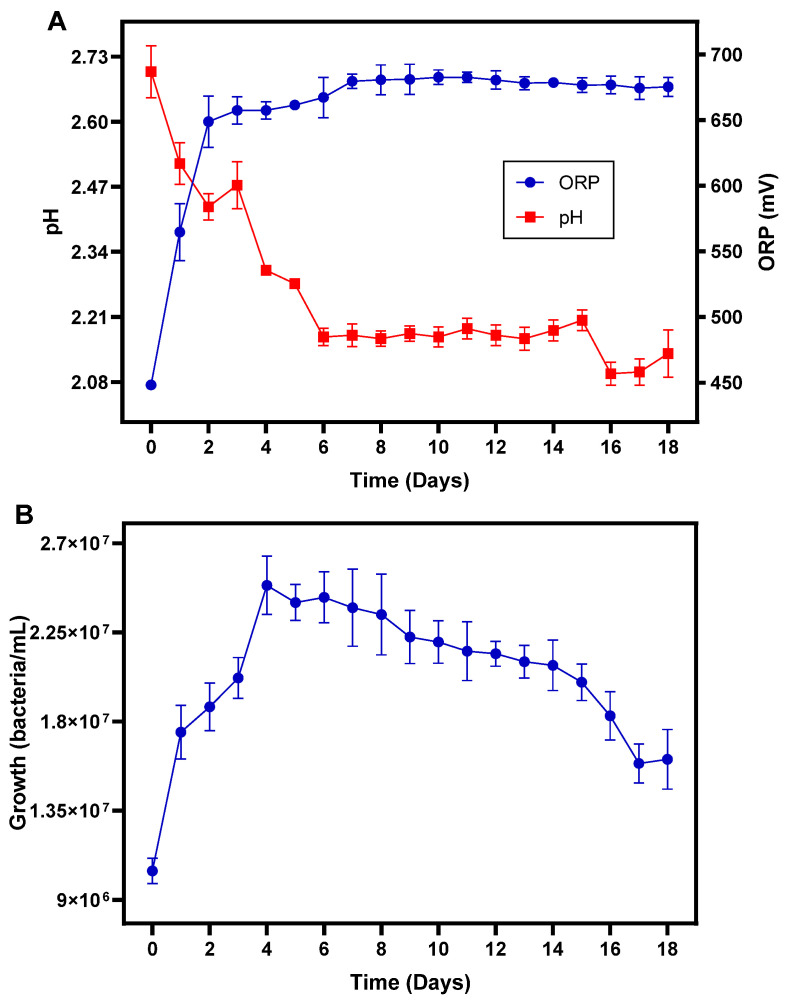
Variation of (**A**) pH and oxidation-reduction potential (ORP) and (**B**) growth of the bacterial consortium, during the bioleaching of PCBs waste.

The ORP is the most important parameter that reflects the extension of oxidation and reduction reactions in the culture medium during the bioleaching of PCBs waste with, generally, high ORP values indicating better oxidation of metals [67]. In an acid culture, medium oxidation occurs by ferric ions (Fe^3+^) of Cu, Zn, gold and tin composing PCBs waste results in iron reduction (Equations (3)–(6)) and in the decreasing of the ORP of the solution, while oxidation of ferrous ions (Fe^2+^) to ferric ions (Fe^3+^) by the bacterial consortium leads to an increase of the ORP of the leaching solution [22,42]. Therefore, the ORP in the solution is the result of metal oxidation, microbial activity and leaching conditions such as aeration (O_2_ and CO_2_), pH, agitation and temperature [46]. Figure 7A shows the evolution of ORP in the bioreactors supplemented with 10 g/L of PCBs waste. The powder from PCBs waste had an immediate effect on the ORP at the beginning of the process (Table 2). Before supplementing the microbial cultures present in the bioreactors with PCBs powder, the ORP was 670.35 mV as a result of the oxidation of the ferrous iron (Figure 6B), however, after supplementing with 10 g/L of PCBs powder the ORP dropped sharply to 448.17 mV (Table 2) due to the alkaline components present in the PCBs. After 2 days of growth with PCBs, the ORP increased rapidly to 648.9 mV due to the oxidation of ferrous ions (Fe^2+^) to ferric ions (Fe^3+^) by the action of the acidophilic iron-oxidizing bacterial consortium, achieving a steady value after day 8, between 675–780 mV. The time required to reach the maximum value of ORP, 682.73 mV, was 10 days, similar to the value obtained by Xia et al. [46] of 699 mV with a consortium formed by *Leptospirillum ferriphilum* and *Acidithiobacillus caldus*. Nonetheless, lower increases in the ORP resultant from the bioleaching process were also reported. For example, Willner et al. [61] showed an increase in ORP between 400 and 550 mV after 14 days of bioleaching with *Acidithiobacillus* *ferrooxidans*. In terms of growth, the consortium reached its maximum growth on day 4 with 2.5 × 10^7^ bacteria/mL (Figure 7B). 

The variation of pH in the culture medium usually reflects the bacterial activity and for the normal growth of acidophilic iron-oxidizing bacteria, the optimum pH should lie between 1.8 and 2.5 [67]). The effect of the powder of PCBs waste on acid production during the bioleaching process is shown in Table 2 and in Figure 7B. The initial bacterial density could have been affected by the strong increase in the pH to 2.7 at the beginning of the bioleaching experiment. This increase in pH may have resulted from the alkaline compounds found in the PCBs waste, which were consumed due to the acidic action of the culture medium. Furthermore, iron was the most abundant metallic component in the leaching solution and the bacterial consortium oxidizes ferrous ions (Fe^2+^) to ferric ions (Fe^3+^) using protons, which also increases the pH value, as shown in Equation (10) [42,67]. Therefore, when starting the leaching experiment, the pH value had to be corrected a couple of times using sulfuric acid (98%) to avoid damage to the bacterial culture and maintain the pH value within the optimal range for the growth of the acidophilic iron-oxidizing bacteria. After 2 days of growth, the pH decreased from 2.7 to 2.4 due to the hydrolysis of ferric ions in the leaching solution (Equations (11) and (12)), generating H^+^. After 6 days, the pH value continued to decrease until it stabilized between 2.09–2.17 until the end of the bioleaching experiment. A similar result was obtained by Wei et al. [21], who observed in a bioleaching experiment with *Acidithiobacillus* *ferrooxidans* a sharp increase in pH from 2.0 to 3.3–3.5 during the first day, after which pH decreased until day 6 to values between 2.3–2.5. However, opposite results were also reported, such as those of Gu et al. [67] that showed a rapid increase in pH from day 1 to 7, reaching a maximum value of approximately 3.5.

4Fe^2+^ + O_2_ + 4H^+^ → 4Fe^3+^ + 2H_2_O (10)

Fe^3+^ + 2H_2_O → Fe(OH)_2_^+^ + 2H^+^ (11)

Fe^3+^ + 3H_2_O → Fe(OH)_3_ + 3H^+^ (12)

## 4. Conclusions

The present study shows the bioleaching of Cu, Zn, tin, lead, gold and silver from PCBs waste by an acidophilic iron-oxidizing bacterial consortium enriched from a sample collected in a gold mine in the Arequipa region, Peru. The enrichment process favorably selected iron-utilizing microorganisms, among which bacteria belonging to the *Leptospirillum* genus had a high prevalence. The Cu and Zn IC were 33 and 38 g/L, respectively; the tolerable maximum concentration for Cu and Zn was 28 and 33 g/L, respectively, with the TI of 0.040 and 0.034, indicating a high capacity of the microbial consortium to tolerate heavy metals. The enriched consortium was preadapted to PCBs waste and showed the best growth up to 10 g/L of this residue, achieving a number of cells of 1.2 × 10^7^ bacteria/mL and a TI of 0.383. TI decreased with the increase of PCB waste. The bioleaching process of 10 g/L of PCBs, carried out in stirred tank reactors coupled to an aeration system, revealed maximum extraction efficiency for Cu and Zn of 68.55% and 91.36%, respectively, with the bioleaching reactors showing a high efficiency for Zn extraction. Lower extraction performances were obtained for tin and gold, being attained values of 15.61% and 27.90%, respectively, and the recovery efficiency of lead and silver was below 0.25%. So the bacterial consortium obtained from the Arequipa region has high efficiency of metal recovery, especially Zn and Cu, which together with the stirred tank reactors (operated at 150 rpm and at 30 ± 1 °C) allowed a greater efficiency of recovery of metals of economic importance. Future work should prioritize the optimization of effective methods for the segregation and recovery of the leached elements based on conventional methods, such as adsorption or electrodeposition.

## Figures and Tables

**Table 1:** Chemical analysis of metals concentration of PCBs (%).

METAL	% in PCBs	METAL	% in PCBs
Cu	10.63	Pb	0.178
Al	>1	Mg	0.154
Ba	>1	Zn	0.131
Ca	>1	Sr	0.072
Fe	0.932	Ag	0.067
Sn	0.899	K	0.053
Ti	0.615	Mn	0.028
B	0.605	Cr	0.017
Ni	0.400	Au	0.009

**Table 2:** Extraction efficiency of heavy metals from PCBs waste in stirred tank bioreactors and evaluation of ORP and pH.

Time (Days)	METAL TYPE (%)				ORP	pH
	Cu	Zn	Au	Sn		
	±SD	±SD	±SD	±SD	±SD	±SD
0	0	0	0	0	* 670 ± 2.1 448 ± 2.4	* 2.2 ± 0.03 2.7 ± 0.05
3	65 ± 0.4	59 ± 3.4	19 ± 0.6	16 ± 5.7	658 ± 10.6	2.5 ± 0.05
7	67 ± 1.8	58 ± 9.7	20 ± 0.0	8 ± 5.3	680 ± 5.6	2.2 ± 0.02
11	69 ± 1.0	70 ± 1.3	28 ± 1.7	7 ± 4.3	683 ± 4.0	2.2 ± 0.02
15	68 ± 1.4	91 ± 1.8	22 ± 1.7	8 ± 5.2	677 ± 5.6	2.2 ± 0.02

Mean, SD: standard deviation, * bacterial growth without PCBs.

## Data Availability

The data presented in this study are available on request from the corresponding author.
